# Blindness

**Published:** 1915-02

**Authors:** 


**Blindness.** Almost all blindness comes at or after birth; there is very little that is hereditary. Of 70,000 cases in the United States, 35,000 were from ophthalmia, in the new born (almost the cruelest thing in all existence, besides being so easily preventable); 21,000 resulted from neglect and abuse; 12 from industrial accidents; and above 600 from our erstwhile insane Independence Day celebrations.—Critic & Guide, January, 1915.

